# Efficacy of Various Laser Wavelengths in the Surgical Treatment of Ankyloglossia: A Systematic Review

**DOI:** 10.3390/life12040558

**Published:** 2022-04-08

**Authors:** Iwona Murias, Kinga Grzech-Leśniak, Anna Murias, Katarzyna Walicka-Cupryś, Marzena Dominiak, Janina Golob Deeb, Jacek Matys

**Affiliations:** 1 EMDOLA, Wroclaw Medical University, 50-425 Wroclaw, Poland; mac-mur@o2.pl; 2 Laser Laboratory, Oral Surgery Department, Wroclaw Medical University, 50-425 Wroclaw, Poland; marzena.dominiak@umed.wroc.pl (M.D.); jacek.matys@wp.pl (J.M.); 3 Department of Periodontics, School of Dentistry, Virginia Commonwealth University, Richmond, VA 23298, USA; jgolobdeeb@vcu.edu; 4 Faculty of Medicine, Pavel Jozef Šafárik University, Trieda SNP 1, 040 11 Košice, Slovakia; anna.maria.murias@student.upjs.sk; 5 Institute of Health Sciences, Faculty of Medicine, University of Rzeszow, 35-959 Rzeszow, Poland; kwcuprys@o2.pl

**Keywords:** ankyloglossia, tongue-tie, short lingual frenulum, lingual frenectomy, lingual frenotomy, lingual frenulectomy, lingual frenulotomy, laser

## Abstract

Ankyloglossia, commonly known as tongue-tie, is the most common disorder of tongue morphology characterized by aberrant attachment of the lingual frenum. This study aimed to provide a comprehensive literature review and evaluate the effectiveness of various laser wavelengths in the surgical treatment of patients with ankyloglossia. An electronic screening of PubMed and the Cochrane Central Register of Controlled Trials (CENTRAL) databases was conducted on 8 November 2021. The following search terms were used to review the available data on the subject of interest: (ankyloglossia OR tongue tie OR short lingual frenulum OR lingual frenectomy OR lingual frenulectomy OR lingual frenotomy OR lingual frenulotomy) AND laser. The use of lasers in ankyloglossia treatment resulted in shorter procedure time, reduced indications for general anesthesia, reduced administration of postoperative analgesics, fewer sutures or none needed, reduced postoperative bleeding, and improved healing. Despite many advantages, this method has its clinical limitations: it requires the use of expensive equipment; well-trained personnel familiar with lasers; and personal protective equipment for the patient, caregiver, operator, and assistant. The laser procedure does not eliminate the need for myofunctional exercises and work with a speech therapist.

## 1. Introduction

Ankyloglossia, commonly known as tongue-tie, is the most common disorder of tongue morphology characterized by improperly attached (too short or thickened) frenulum of the tongue. The lingual frenulum is formed by the dynamic elevation of a midline fold in the floor of the mouth fascia. This diaphragm-like structure suspends the tongue and the floor of mouth structures within the arc of the mandible, creating a balance between mobility and stability [1,2,3]. Type I and III collagen fibers and type III elastin fibers constitute a large share in the structure of the tongue frenulum [1]. Ankyloglossia can impair breastfeeding (nipple pain, poor infant weight gain, and early weaning), speech, swallowing, breathing, sleep, and oral hygiene. Furthermore, it can cause oral dysfunction, social problems related to impaired language function, and even postural problems [4,5,6,7,8,9,10,11]. The incidence of ankyloglossia reported in the literature ranges from 0.02% to 10.7% [4,12,13,14,15,16]. This discrepancy, in part, may be related to different assessment methods and classifications used to diagnose this disease entity. Various studies reported the clinical significance of ankyloglossia as a cause of breastfeeding difficulties, sore nipples, inadequate infant weight gain, neonatal dehydration, and shortened breastfeeding duration [17,18,19]. Moreover, the National Institute for Health and Clinical Excellence (NICE) approved and recommended, for healthcare professionals, the surgical ankyloglossia treatment of children with tongue-tie [17].

The scientific literature shows numerous classifications of ankyloglossia [20,21,22,23,24,25,26,27]. One of the most recognized is Kotlow classification [20], which assesses the length of the free part of the tongue and measures the distance from the tip of the tongue to the place of attachment of the bit on the tongue. Another, the Coryllos classification [21], describes the appearance of the frenulum and attachment site. The classification of Todd–Hogan shows division into anterior and posterior frenulum; in turn, Ruffoli classification [22] measures the length of the bit from the bottom of the mouth to the attachment point on the tongue, and the maximum opening of the mouth with the tongue on the incisive nipple. The functional classifications were described by Hazelbaker [23], Amir [24] Martinelli [25], and Marchesan [26,27]. Among the listed, Marchesan described the clinically useful classification that measures the difference (given as a percentage) between the maximum mouth opening with the tongue resting at the bottom of the mouth and with the tongue touching the palatal papilla.

Curing ankyloglossia is achieved mainly through surgical intervention performed within the structures that build the tongue frenulum, most often in combination with myofunctional exercises performed before and after the procedure [5,9,28]. Other complementary medical treatments include craniosacral therapy, orofacial myofunctional therapy, chiropractic care, and naturopathy. The scientific literature is scarce on high-quality research on the effectiveness of these techniques [11].

The most common surgical procedures for treatment of ankyloglossia are frenotomy, frenectomy, and frenuloplasty (Z-plasty) [5,29]. Treatment of ankyloglossia can be performed using the conventional method using scissors/scalpel, electrocautery, or laser [4,6,30].

Various types of lasers can be used in the treatment of ankyloglossia; however, the laser wavelength selection should be based on optical affinity for hemoglobin and water. Different lasers were used in studies for tongue frenulum surgery, e.g., diode lasers [9,10,28,31,32,33,34,35,36,37,38,39,40], erbium family lasers [4,39,41], carbon dioxide (CO_2_) lasers [42,43,44,45,46], neodymium lasers [47,48], and potassium titanyl phosphate (KTP) lasers [49].

Diode and neodymium lasers enable precise cutting, provide hemostasis, and have biomodulating properties but can thermally damage surrounding tissues [50,51,52,53,54]. The chromophore of CO_2_ lasers is hydroxyapatite and water. These lasers enable precise cutting with a simultaneous hemostatic effect due to the thermal effect. They have the ability to close small blood and lymph vessels, have anti-inflammatory properties, cause soft tissue carbonization, and are frequently used in treatment of larger tissue areas [55,56]. Erbium family lasers, including Erbium-doped Yttrium Aluminum Garnet laser (Er:YAG), and Erbium, Chromium-doped Yttrium, Scandium, Gallium, and Garnet (Er,Cr:YSGG), have high affinity for water and lower hemostatic capacity, compared to other laser wavelengths [54,57,58,59].

This study aimed to provide a comprehensive literature review and evaluate the effectiveness of various laser wavelengths in the surgical treatment of patients with ankyloglossia.

## 2. Materials and Methods

### 2.1. Focused Question

The focused question in the paper was: ”Is the use of lasers in the surgical treatment of ankyloglossia in patients’ effective method?”

### 2.2. Protocol

**Figure 1:**
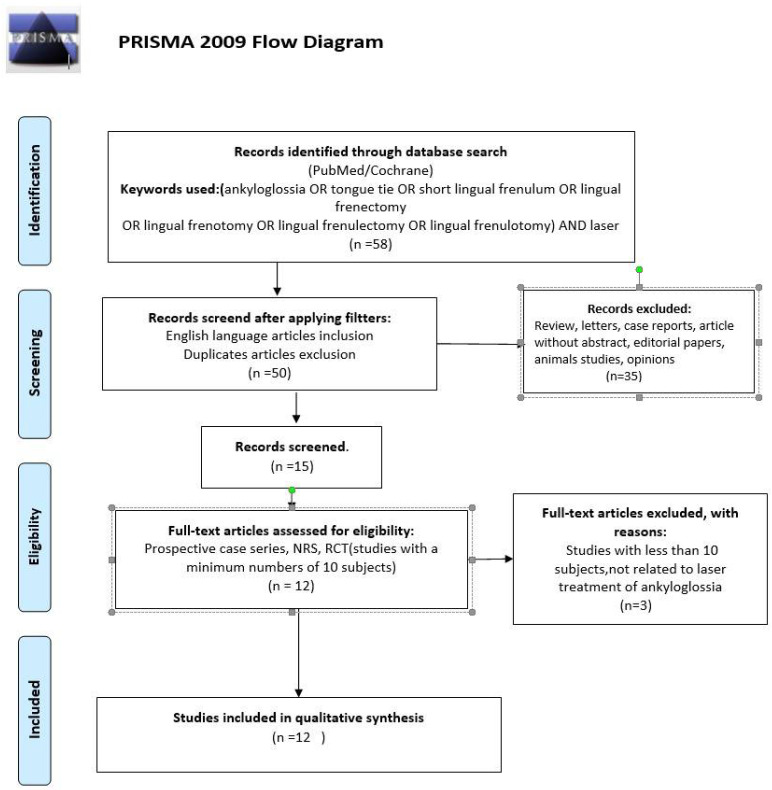
PRISMA flowchart presenting the criteria for the included studies.

The review was prepared in accordance with the PRISMA statement [60], as well as the Cochrane Handbook of Systematic Reviews of Interventions [61]. Details of the selection criteria are presented in Figure 1.

### 2.3. Eligibility Criteria

Studies were considered acceptable for inclusion in the review if they fulfilled the following criteria:

- Studies involving human subjects;
- Surgical use of dental lasers in the treatment of ankyloglossia;
- Studies in English language;
- Clinical reports amounted to 10 or more cases;
- Non-randomized controlled clinical trials (NRS);
- Randomized controlled clinical trials (RCT).

The exclusion criteria the reviewers agreed upon were as follows:

- Non-English papers;
- Opinions;
- Letters to the editor;
- Editorial papers;
- Review articles;
- Clinical reports with fewer than 10 cases;
- No full-text accessible;
- Duplicated publications;
- Treatment of ankyloglossia without laser.

No restrictions were applied concerning the year of publication.

### 2.4. Information Sources, Search Strategy, and Study Selection

An electronic screening of PubMed and the Cochrane Central Register of Controlled Trials (CENTRAL) databases was conducted on 8 November 2021. To review the data available on the subject of interest, the following search terms were used: (ankyloglossia OR tongue-tie OR short lingual frenulum OR lingual frenectomy OR lingual frenulectomy OR lingual frenotomy OR lingual frenulotomy) AND laser. The search was limited to human subjects and studies with other eligibility criteria. The references of all selected full-text papers and related reviews were screened. Only articles with available or accessible full-text versions were considered. An attempt was made to contact the corresponding authors of unpublished or missing data if needed.

### 2.5. Data Collection Process, Data Items

Study selection was conducted in two phases. Four reviewers independently extracted data from articles that met the inclusion criteria (IM, AM, JM, and KGL). Articles marked for inclusion by at least two reviewers were retained for the second phase of full-text review. Two reviewers examined all full-text articles independently (IM, AM), and two reviewers (KGL, JM) were consulted to resolve any disagreements. The data used were as follows: first author, year of publication, title, study design, study groups, study results (number of study groups, age of respondents, presence of myofunctional and speech therapy recommendations before after surgery, suturing after surgery, the type of anesthesia used, laser type, and laser parameters). Extracted data were entered into a standardized Excel file.

### 2.6. Risk of Bias in Individual Studies

In the initial study selection, to minimize the potential for reviewer bias, each author screened titles and abstracts independently. The Cohen k test determined the level of agreement between reviewers [62]. Discussion between the authors resolved any difference in opinion on the inclusion or exclusion of a study.

### 2.7. Quality Assessment

The methodological quality of each of the included studies was assessed by two reviewers working independently. The criteria on which the project, implementation, and analysis are based are as follows:

- Randomization (1) or its absence (0);
- Group size of at least 10 subjects (1) or its absence (0);
- Given laser parameters, e.g., wavelength, power, energy density, frequency, and applicator (1), or its absence (0);
- Assessment of the functions of the tongue before and after the procedure (1) or its absence (0);
- Recommended myofunctional therapy (1) or its absence (0);
- Presence of a control group without a laser (1) or its absence (0);
- Minimum 3-month observation period (1) or its absence (0);
- Description of the technique of the performed procedure, detailed information, e.g., additional instruments supporting the procedure, duration of the procedure (1), or its absence (0);
- A study containing a simple size (1) or its absence (0).

The descriptive information about the studies was graded. Studies were scored on a scale from zero to nine points (score 0–3 low, 4–6 moderate, and 7–9 high quality of a study). Any disagreements were resolved through discussion until reaching a consensus.

### 2.8. Risk of Bias across Studies

After summing up the results of each study, an overall calculation of the risk of bias (low, moderate, or high) for each publication, as recommended by the Cochrane Handbook for Systematic Reviews of Interventions, was performed [61].

## 3. Results

### 3.1. Study Selection

Initially, 58 studies were identified as subject to analysis. After screening the titles and abstracts, 34 studies were excluded. Fifteen studies were selected for further full-text analysis, from which three were excluded according to predefined inclusion [63,64,65]. The reasons for exclusion were described in Table 1.

Finally, twelve publications were included in the review [4,9,10,28,34,36,42,43,44,45,66,67].

### 3.2. General Characteristics of the Included Studies

Of the twelve studies included in the review, two were randomized controlled trials [9,66], one was a pilot study [36], and nine were prospective case series [4,10,28,34,42,43,44,45,67]. Various types of lasers have been used in studies to treat ankyloglossia. Diode lasers were used in six studies [9,10,28,34,36,66], a CO_2_ laser was used in five studies [42,43,44,45,67], and an Er:YAG laser was used in two studies [4,66]. Two different laser wavelengths were used in two studies: two diode lasers [34] and the diode and Er:YAG laser [66]. Detailed information on the parameters of the lasers used was provided in seven studies [4,9,10,34,43,45,67]. Various methods of anesthesia were used before the procedure. Local anesthesia was used in seven studies [4,9,28,43,44,45,66,67], only topical anesthesia was used in one study [10], and no information about the anesthesia was reported in two manuscripts [34,36]. In one study, local anesthesia was used as optional and not for each patient [66], one study used general or local anesthesia optionally [42], and one study used both topical and local anesthesia [45].

The age of the subjects included early childhood, preschool, and school-aged children in seven studies [4,9,28,42,43,44,45], two studies included only a group of newborns and infants [4,34], two studies included children and adults with an extensive age range of respondents [36,67], and one study included only adults [66]. As for the type of laser used, the one study with early childhood and school-aged children used Er:YAG laser [4], two scientific experiments with newborns and infants used diode lasers [10,34], and one study involving adults used two different lasers (diode and Er:YAG) [66]. The general characteristics of the included studies are presented in Table 2.

### 3.3. Subjects of the Study

Studies included in the review were evaluated for the type of laser and its correlation with the use of analgesics, antibiotics, and postoperative sutures, for treatment of ankyloglossia. After analyzing research, the following information was obtained: analgesics were administrated in three studies using CO_2_ lasers [43,45,67] and in one using a diode laser [10]. Analgesics with antibiotics were used in one study by Komori et al. [42]. No data were available regarding the use of antibiotics and/or analgesics in the articles with diode [9,28,34,36,66] and Er:YAG [4,66] lasers. Sutures were only needed for eight patients who received treatment for ankyloglossia with a CO_2_ [42] and Er:YAG laser [4] (for available articles see Table 3).

Most of the included studies recommended myofunctional therapy after surgical ankyloglossia treatment [4,9,10,28,36,42,43,45]. In two studies including newborns and infants, standardized survey questionnaires were used to evaluate subjects at the same follow-up period (1 month) [10,34]. Both of these studies reported a significant improvement in language functions. In six studies where the age groups were not homogeneous, different methods and classifications were used to assess the improvement of tongue functions. The observation periods in these studies also varied significantly, ranging from 1 day to 12 months [4,9,28,36,43,67]. Eight studies reported significant improvement in language functions [4,9,10,28,34,36,43,67]. Post-operative tongue functions were not assessed in four studies [42,44,45,66]. A significant improvement in body posture following laser treatment of ankyloglossia was observed by Olivi et al. [4]. One study reported an improvement in obstructive sleep apnea symptoms [9] (for available articles see Table 4).

### 3.4. Quality Assessment and Risk of Bias across Studies

The paper by Fioravanti et al. [9] included in the review was qualified as high-quality scoring. Nine articles were scored as moderate-quality [4,10,28,34,42,43,45,66,67]. Two articles were obtained as low-quality (high risk of bias) [36,44]. Quality assessment and risk of bias of the included studies were described in Table 5.

## 4. Discussion

Twelve studies met the inclusion criteria and demonstrated the effectiveness of laser surgery in treatment of ankyloglossia [4,9,10,28,34,36,42,43,44,45,66,67]. Most of the studies included in the review showed improvements in tongue function and increased tongue length after laser treatment [4,9,10,28,36,42,43,45]. Two studies investigated the Er: YAG laser [4,66], but only Olivi et al. [4] employed the Er: YAG laser for treatment of ankyloglossia in children aged 8 to 18 years. The surgical treatment of ankyloglossia, when using the CO_2_ laser [42,43,44,45,67], and various wavelengths of diode laser [9,10,28,34,36,66] are widely described in scientific literature. The considerable advantages of using lasers in treating ankyloglossia include improvement in tongue length [4,9,10,28,34,36,42,43,44,45,66,67], improvement in speech functions [4,9,10,28,36,42,43,45], and a reduced need for antibiotics, analgesics, and sutures in most patients.

The authors of qualified studies recommend administering local infiltration anesthesia in the sublingual area before the procedure [4,9,28,42,43,44,45,66,67]. Some authors point out that in the case of laser procedures, the amount of anesthetic administered can be significantly reduced [4,42,44]. The use of only topical anesthesia before the procedure was recommended by Ghaheri et al. [10] and Fiorotti et al. [45]. Apart from topical anesthesia, Aras et al. [66] suggested the use of infiltration anesthesia, depending on the type of laser used for the procedure (in the case of a diode laser, additional infiltration anesthesia was recommended, while for Er:YAG it was was optional). Baxter et al. [43] emphasized the possibility of using additional sedation with nitrous oxide, if necessary, while Komori et al. [42] recommended that children under three years of age should undergo the procedure under general anesthesia. The overwhelming majority of authors [9,10,28,34,36,43,44,45,66,67] did not report the need for suturing after ankyloglossia treatment with lasers. In only one case, among patients in the Olivi et al. [4] study, suturing was needed to improve haemostasis. Additionally, Komori et al. [42] trial required suturing in 7 of 15 patients. The use of lasers for ankyloglossia treatment indicates the simplification of surgical treatment, especially when this procedure is performed on children, where the shortening of the procedure time is beneficial for the patient.

Most of the authors of the qualified studies note the need for myofunctional therapy in patients undergoing laser ankyloglossia treatment [4,9,10,28,36,42,43,45]. In studies involving infants and very young children, the implementation of myofunctional therapy was entrusted to the caregivers of children and involved the immediate postoperative period [10,42,43,45]. Baxter et al. [43], for the children aged 13 months to 13 years, recommended to manually stretch the wound 2 to 3 times per day. Komori et al. [42] and Fiorotti et al. [45] recommended tongue extension exercises for the children aged below 15 years. For newborns and infants aged 0–12 weeks, Ghaheri et al. [10] recommended a very detailed set of myofunctional exercises that gently elevate the tongue and massage the wound four to six times per day for several weeks. Myofunctional therapy, as an adjunctive to surgical treatment of ancyloglosia, significantly improved the mobility and functions of the tongue [4,9]. However, it should be highlighted that due to the different research methodologies and ages of the children, as well as the differences in the recommended myofunctional exercises, including duration, frequency, and the period after which the comparative assessment was made, it is difficult to clearly state which model of recommended myofunctional therapy is the most beneficial for patients. It is certain, however, that the use of myofunctional therapy simultaneously with the surgical treatment of ankyloglossia can significantly improve the tongue function [4,9,10,28,36,42,43,45].

Another important issue in the qualified studies concerns the postoperative administration of analgesics and the healing process after laser treatment of ankyloglossia. The recommendations regarding the use of analgesics and antibiotics involve preferably limiting or excluding the use of the aforementioned drugs. Several of the qualified studies did not provide any information on the use of drugs following laser surgery [4,9,28,36,44,66]. The administration of analgesics has been reported in four studies [10,43,45,67]. Four authors [10,43,45,67] reported the administration of acetaminophen for the first one to two days as needed, but the majority of infants needed little, if any, analgesia post procedure. Neither author reported impaired healing of a surgical wound after ankyloglossia surgery. The only reported complication was the reattachment of the frenum, found only in one case of the 15 subjects in the study described by Komori et al. [42]. Additionally, in the study of Olive et al. [4] the authors reported a complication in the form of bleeding immediately after the laser surgery in the one of 29 patients.

Last but not least, in two studies, apart from the improvement of tongue function, an attempt was made to assess body posture after laser treatment on the lingual frenulum [4,36]. However, these studies differ in the period of postoperative follow-up. Saccomanno et al. [36] performed an immediate postoperative examination by spirometry and observed no significant postural improvement. Olivi et al. [4] performed a body posture assessment before and two months after the procedure using a proprietary device and observed significant postural improvement in 18 of 30 patients. Further studies should be conducted to examine whether laser-assisted ankyloglossia surgery combined with myofunctional therapy improves body posture and speech function in children.

## 5. Conclusions

The use of lasers for the surgical treatment of ankyloglossia seems to be an effective and promising method for children and adults. The employment of lasers offers several benefits, including shorter procedural time, reduced use of general and local anesthesia and suturing, a reduced need for the administration of analgesic and/or anti-inflammatory drugs, favorable postoperative healing, and a reduction in postoperative bleeding and complications (in the form of inflammation or hematomas).

Despite its many advantages, this method has its clinical limitations: it requires the use of expensive equipment; well-trained personnel familiar with laser handling and operation; and the use of personal protective equipment for the patient, caregiver, operator, and assistant. The laser procedure does not eliminate the need for myofunctional exercises and work with a speech therapist.

## Figures and Tables

**Table 1:** Reasons for exclusion of studies.

Ordinar Number	Reason for Exclusion	Reference Number
1	Only seven subjects	Favero et al. [63]
2	Does not apply to ankyloglossia	Kotlow et al. [64]
3	Laser used for acupuncture	El-Bassyouni et al. [65]

**Table 2:** General characteristics of the included studies.

First Author	Study Type	Patients Age	Laser Type and Parameters	Type of Anesthesia
Tripodi et al. [28]	Prospective case series, two groups: 90 test group (laser and speech therapy) and 90 control group (laser without speech therapy)	6–12 years	Diode 4 W Fiber 320 μm	Local anesthesia (articaine with epinephrine) 1:200,000)
Baxter et al. [43]	Prospective case series: one group; 3 subjects without control group, 23 with a tongue-tie released, and 14 tongue-tie and lip-tie	13 months- 13 years	CO_2_ 2 W, 29 Hz, 72.5% duty Non-SuperPulse	Local anesthesia (2% lidocaine with 1:100,000 epinephrine)
Hand et al. [34]	Prospective case series: one group (130 subjects tongue -tie), without control group; 82 subjects—laser 1470 nm; and 50 subjects—laser 980 nm	Mean age 43 days	Diode lasers: 1470 nm and 980 nm 1470 nm: 3.5 W, 50 ms Ton, and 50 ms Toff, Fibre 300 μm 980 nm: 4.0 W, 100 μs Ton/Toff 100 μs, and Fibre 300 μm	No information
Olivi et al. [4]	Prospective case series: one group, 29 subjects, without control group	8–18 years	Er:YAG Energy 120–160 mJ, tip 600 μm, lengths (9–14 mm), and 15 Hz Pulse duration 600–300 μs water spray (10 mL/min)	Local anesthesia (4% articaine with epinephrine 1:200,000, 0.6 mL)
Ghaheri et al. [10]	Prospective case series: one group, 236 subjects, tongue-tie and lip-tie	0–12 weeks	Diode 1064 nm 0.47–0.53 W, 200 us Ton, 100 us Tof, and Fibre 300 μm	Topical anesthesia EMLA
Komori et al. [42]	Prospective case series one group, 15 subjects tongue-tie 6 subjects lip-tie, without control group	Mean age 5, 2 years	CO_2_, 2–5 W	General anesthesia or Local anesthesia
Arras et al. [66]	RCT, 16 subjects, 2 groups, 8 per group	18–27 years	Er:YAG and Diode 808 nm 1 W, 2 W	Local anesthesia (optional) 2 mL articaine hydrochloride
Saccomanno et al. [36]	Prospective case series: (Pilot Study) one group, 24 subjects, without control group	10–26 years	Diode 660 nm Lack of information	No information
Puthussery et al. [67]	Prospective case series: one group, 21 subjects, without control group	3–30 years	CO_2_ 4 W, 25 J/cm^2^ continuous mode	Local anesthesia with 2% lidocaine and 1:80,000 epinephrine
Kato et al. [44]	Prospective case series: one group, 20 subjects with tongue-tie, without control group	1–15 years	CO_2_ 3 W, tip 1 mm diameter	Local anesthesia
Fioravanti et al. [9]	RCT: 32 subjects, 2 groups, and 16 per group 1—Laser and myofunctional therapy 2—myofunctional therapy	4–13 years	Diode 980 nm Peak power 7.5 W Frequency up to 25 Hz Continuous mode	Local anesthesia
Fiorotti et al. [45]	Prospective case series: 1 group, 15 subjects, without control group	2–15 years	CO_2_ 6 W, Intensity 191 W/cm^2^	Topically preanesthetized 10% Lidocaine spray 1.8 mL of anesthetic solution (2% lidocaine without a vasoconstrictor)

**Table 3:** The use of analgesics, antibiotics, and sutures across included studies.

	CO_2_ Lasers	Diode Lasers	Er:YAG Lasers
Use of analgesics	Baxter et al. [43] Fiorotti et al. [45] Puthussery et al. [67]	Ghaheri et al. [10]	-
Use of analgesics and antibiotics	Komori et al. [42]	-	-
No information about the medications used	Kato et al. [44]	Tripodi et al. [28] Hang et al. [34] Saccomanno et al. [36] Fioravanti et al. [9] Aras et al. [66]	Olivi et al. [4] Aras et al. [66]
Use of sutures	Komori et al. (7 out of 15 subjects) [42]	-	Olivi et al. (1 subject) [4]

**Table 4:** Characteristics of methods for assessing the improvement of tongue functions, observation period, applied myofunctional therapy, and age of the patients.

First Author	Myofunctional Therapy	Length of the Observation Process	Evaluation of Functions or/and Measurements of Frenulum, Pain, Wound Healing, Body Posture	Recorded Improvement	Age of the Respondents
Tripodi et al. [28]	Speech therapy protocol rehabilitation 3 months	12 months	Pre and post intervention Ruffoli’s classification of the lingual frenulum length. Pre-surgery and post-surgery at 1 week, 1 month, 3 months, 6 months, and 12 months follow-up.	Significant improvement in the mean values of the maximum mouth opening	6–12 years
Baxter et al. [43]	Myofunctional exercises were recommended. Manual stretching of the wound 2 to 3 times daily	1 month	Pre and post intervention Parents reported improvementof speech, feeding, and sleep; 1 week in person follow-up, 1 month in person or phone follow-up	Significant improvement in speech, feeding, and sleep	13 months– 13 years
Hand et al. [34]	Without myofunctional therapy	1 month	Pre and post intervention at 1 week and 1 month post-operative surveys folllow-up Revised Infant Gastroesophageal Reflux Questionnaire (I-GERQ-R), visual analogue scale (VAS) for severity of nipple pain, and Breastfeeding Self-Efficacy Scale- Short Form	Statistically significant improvement in: I-GERQ-R, BSES-SF, and VAS pain-breastfeeding	Mean age 43 days
Olivi et al. [4]	Myofunctional exercises were recom- mended	2 months	Pre and post intervention at 7 days follow-up control of healing; 21 days follow-up tongue movement functions; and 2 months follow-up tongue functions and body posture	Improvement lingual movement and functions 2-months improvement lingual functions and significant postural improvement on 18 of 30 patients	8–18 years
Ghaheri et al. [10]	Myofunctional exercises were recommended. Postprocedural stretching exercises were advised to avoid reattachment of tissue by gently elevating the tongue and massaging the wound four to six times per day for several weeks	1 month	Pre and post intervention at 1 week and 1 month follow-up Suction assessment Head and neck assessment or there are limitations, interview, Coryllos scale for tongue, BSES-SF, I-GERQ-R, and VAS pain	Significant improvement in: BSES-SF, I-GERQ-R, and VAS pain-breastfeeding	0–12 weeks
Komori et al. [42]	Myofunctional exercises were recommended tongue extension exercises	Observation protocol non-uniform Observation period patients after surgery from 1 week to 3 years	Pre-intervention Ito classification (3 levels) Interview with parent Evaluation of speech, eating, and sucking disorders		1 month-14 years mean age 5, 2 years
Aras et al. [66]	No information	1 week	One day and one week after surgery, Assessment of pain and oral function: chewing, eating, and speaking. Oral function were assessed with a 5-point Likert scale. Pain levels were assessed with a 7-point Likert-type scale		18–27 years
Saccomanno et al. [36]	Myofunctional exercises were recommended myofunctional protocol requires the exercises to be repeated 3 times a day for 15 min a day (5 min × 3) for 1 month before and 3 months after the surgery	1 day	Pre and post intervention Marchesan protocol and spirometry	No statistically significant improvement	10–26 years
Puthussery et al. [67]	No information	1 month	Pre and post intervention, 1 and 7 days, 1 month after surgery. Assessments of pain, swelling, bleeding, speech, tongue movement, and oral hygiene	Improvement in: speech, tongue movement, and oral hygiene	3–30 years
Kato et al. [44]	No information	2 weeks	Evaluation of wound healing 1, 2, and 3 weeks after surgery		1–15 years
Fioravanti et al. [9]	Myofunctional exercises were recommended. Therapy for 3 months, home exercises about 1 h a day	3 months	Pre and post intervention Kotlow and Ruffoli (0 and 28 day) Polysomnography (before and 3 months after)-OSAS assessment Evaluation of the painful symptoms in the days following the surgery at 24 h, 48 h, 72 h, 14 days, and 28 days after surgery	Improvement in: Kotlow, MAB, MOTTIP, and Protrusion Improvement in polysomnography- OSAS syndrome	4–13 years
Fiorotti et al. [45]	Myofunctional exercises were recom mended	15 days	Pre-surgery questionnaire (respiration; difficulties in making speech sounds; damaging oromyofunctional habits (thumb sucking, pacifier sucking); and inadequate postural habits or discomfort during feeding (pacifier sucking).		2–15 years

**Table 5:** Quality assessment and risk of bias of the included studies.

Criteria	First Autor											
	Tripodi et al. [28]	Baxter et al. [43]	Hand et al. [34]	Olivi et al. [4]	Ghaheri et al. [10]	Komori et al. [42]	Aras et al. [66]	Saccomanno et al. [36]	Puthussery et al. [67]	Kato et al. [44]	Fioravanti et al. [9]	Fiorotti et al. [45]
Randomization	0	0	0	0	0	0	1	0	0	0	1	0
Laser type (wavelenght)	1	1	1	1	1	1	1	1	1	1	1	1
Laser parameters: power, energy, density, and applicator type	1	1	1	1	1	1	1	1	1	1	1	1
Myofunctional therapy was prescribed	1	1	0	1	1	1	0	1	0	0	1	1
Function evaluation of the tongue made	1	1	1	1	1	0	1	0	1	0	1	0
Presence of the control group (without a laser)	0	0	0	0	0	0	0	0	0	0	1	0
Presence of at least 3 months observation	1	0	0	1	0	1	0	0	0	0	1	0
Description of the performed procedure and other detailed information, e.g., the use of additional instruments	0	1	1	1	1	0	1	0	1	1	1	1
Presence of Sample Size	0	0	0	0	1	0	0	0	0	0	1	0
Total	5	5	4	6	6	4	5	3	4	3	9	4
Risk of bias	Moderate	Moderate	Moderate	Moderate	Moderate	Moderate	Moderate	High	Moderate	High	Low	Moderate
